# Country Pharmaceutical Situation on Access, Quality, and Rational Use of Medicines: An Evidence from a middle-income country

**DOI:** 10.22037/ijpr.2019.111636.13273

**Published:** 2019

**Authors:** Hossein Minaei, Mohammad Peikanpour, Nazila Yousefi, Payam Peymani, Farzad Peiravian, Nikta Shobeiri, Zahra Karimi Majd, Javad Shamsaee

**Affiliations:** a *Department of Pharmacoeconomics and Pharma Management, School of Pharmacy, Shahid Beheshti University of Medical Sciences, Tehran, Iran. *; b *Health Policy Research Center, Institute of Health, Shiraz University of Medical Science, Shiraz, Iran.*; c *H. M. and M. P. contributed equally to this work.*

**Keywords:** Access, Affordability, Rational use of medicines, Quality, Pharmaceutical policy

## Abstract

Evaluation of pharmaceutical systems performance is an essential prerequisite for promoting evidence-based policy-making and improvement in health system performance. This study attempts to evaluate the performance of Iran pharmaceutical system based on the world health organization (WHO)′s indicators, including access, quality, and rational use of medicines. In this cross-sectional descriptive study, inspired by the instructions proposed by WHO, public and private pharmaceutical service-providers were evaluated in three dimensions and 16 indicators. Accordingly, eleven separate checklists were developed and, in terms of translation, face and content validity were certified by pharmaceutical sector’s experts. Sampling was randomly carried out in five cities. Depending on the type of indicators, retrospective or prospective approaches was determined for data collection. The data were collected from April to November 2018 and analyzed by SPSS 24. The availability of targeted key medicines in various cities as well as in public and private pharmacies was 97.5% with no significant difference. Although the medicines cost was higher in private sectors than in public ones, they were affordable in both sectors. In quality indicators, public sectors enjoyed a higher level than the private sectors did. The average number of medicines per prescription in public pharmacies was 3.2 and it was 3.4 in private ones. On average, in public sectors 33% and 32% of outpatients received antibiotics and injectable medicines, respectively. Finally, 77% of medicines were prescribed by using their generic names and 25% of prescriptions were in accordance with key medicines list. This study reveals that the availability and affordability of targeted key medicines in Iran are in good condition; however, in terms of rational use of medicines, Iran fails to meet the standard levels.

## Introduction

Since pharmaceutical services are of prime importance and play a key role in health system, improving quality services have always been one of policymakers′ main priorities in pharmaceutical sector. Ensuring the quality and safety of medicines, on-time provision of medicines, observing patients′ affordability, and improving rational use of medicines are all among the goals of this system. Policy making in pharmaceutical system necessitates providing scientific evidence drawn from the present conditions and the impacts of previous decisions. The evaluation of pharmaceutical system highlights the effectiveness of previous policies and their positive and negative consequences as well as the need for adopting new policies (1).

Due to the vital role of medicines in controlling burden of diseases and in decreasing their mortality rates, it should be noted that this aim can be achieved when medicines are available to all walks of life. High prices of medicines and their unavailability in pharmacies are the factors which can deteriorate patients′ health. Access generally means medicines are physically available in pharmacies and affordable for all citizens (2). Likewise, according to the laws of health systems in most countries, people are entitled to have access to essential medicines, and governments are to fulfill this aim (3). According to the world health organization (WHO)′s reports, around one-third of people in the world do not have assured access to essential medicines (4); that fifty percent of them live in Africa and Asia (5). In addition, according to WHO recommendations, to warrantee patients’ safety and promote community health level, it is necessary to evaluate pharmaceutical service providers in terms of “quality” (6). Quality is defined as “being free from defects, deficiencies, and significant variations” in service provision (7). As a result, quality, as a commitment to national or international standards, can promote health indicators, increase health system reliability, and enhance patients’ trust to pharmaceutical service providers. Furthermore, in the past decades, development of rational use of medicines was continuously one of the major priorities in pharmaceutical policy-making. According to the WHO′s report more than fifty percent of medicines are prescribed, dispensed, and sold irrationally (8). Economic losses on patients and health system, occurrence of adverse drug reactions, increased medicine resistance, prolonged illness or finally death can be the consequences and harms of irrational use of medicines (9). Rational use of medicines is defined as ″patients should receive medications commensurate with their clinical needs, in appropriate dosage that meet their individual requirements, for an adequate period of time, and at lowest cost to them and their community″ (10). Physicians and other health sectors’ professions, therefore, play the main role in the development of rational use of medicines. The average number of medicines per prescription, the percentage of antibiotics and injectable medicines in prescriptions, medicines prescribed by generic name, and prescription based on essential medicines list as well as standard therapeutic guidelines (STGs) are the main criteria to evaluate rational use of medicines (6).

This study aims to evaluate the performance of Iran′s pharmaceutical service providers to patients in terms of access to key medicines which were selected by research team according to WHO’s recommended list and national clinical practices, quality of drugs, and the status of rational use of medicines accordance with the WHO instructions. Despite the WHO′s advice concerning conducting such periodical evaluations, in Iran, some thinly scattered studies were carried out in each of these dimensions past few years (11, 12), yet the present study covers all the indicators related to these three dimensions all inclusively. The results of this study can indicate whether the aims of pharmaceutical system have been fulfilled or not and can clearly reveal the existing gaps to the policy-makers.

*Methods*

*Study design*

This research has been carried out based on the level II of “WHO′s operational package for assessing, monitoring and evaluating country pharmaceutical situations” (6). Dimensions and indicators investigated in this study are provided in Table 1. In the primary stage of designing study, the checklists had been exactly extracted from WHO guideline (by adjusting some items such as the list of key medicines). However, due to lack of IT capacity in Iran PHFs, missing of medicines’ shortage history recording, and lack of diseases diagnosis records in prescriptions, three survey forms (about average stock out duration, availability of standard treatment guidelines, and tracer cases treated according to recommended treatment protocols) were excluded in this survey. Similarly, three forms which are related to wholesalers’ data were omitted since they are not directly related to the aims of this study, analyzing service providers to end-users. Based on this instruction for evaluating these indicators, eleven separate checklists were developed and were scrutinized and certified by nine pharmaceutical sector experts, in terms of translation, content, and face validity. The experts, with minimum five-years experiences in pharmaceutical policy and management, were invited for face to face interview to validate checklists.

In order to investigate the affordability of medicines, standard treatments of diseases, including diabetes, asthma, hypertension, hyperlipidemia, pulmonary, and out-patient pneumonia were examined. By dividing the patients′ payment for a course of pharmacotherapy or the needed medicines for a one-month treatment of chronic diseases into the daily wage of the lowest paid unskilled government worker (LPGW), the affordability of such periods have been calculated (13). Should the result of such division be less than one (≤1), the purchase of medicines for a treatment period can be affordable. In 2018, the minimum daily wage in Iran was 380000 Rials (9 USD; exchange rate 42000 Rials). Regarding the indicator of geographic accessibility to pharmacies, the percentage of patients who get to a pharmacy by walking within maximum thirty minutes was investigated. 

In order to compare the price of essential medicines, median price ratio (MPR) was considered, which is gained by comparing the price of each medicine with its international price. According to Health Action International (HAI) (14), the source utilized as a reference for exchange rates was the websites of “World Bank”, “IMF”, and “Central Bank of Iran”. According to these references, each U.S dollar was equal to 42000 Iranian Rials. By dividing the price of a given medicine in Iran into its reference price, MPR can be gained. This indicator should be less than 1.5 and 2 in public and private sector, respectively (15).

Concerning the quality, as WHO guideline recommends, the existence of expired medicines in pharmacies and observing the standards of storing medicines were considered as evaluation indicators. Standards concerning storing medicines based on the WHO guideline are: controlling the pharmacy temperature and appropriate thermal insulation, proper ventilation, not being exposed to direct sunlight, controlled humidity of the pharmacy, having air-conditioner and temperature log-sheets, sorting of medicines based on the expiry dates, having pest control program, not manipulation of tablets/capsules by naked hands, and not storing medicines directly on the floor. Since there is no list of essential medicines in Iran, a list of key medicines was prepared so that some indicators such as availability of medicines, the percentage of expired medicines, and the price of key medicines could be provided. The list of key medicines was developed considering the list of 15 medicines recommended by WHO, expanding to 25 medicines based on clinical expert opinion. The 25 selected medicines are prescribed in primary and secondary health care levels for common diseases and such medicines are expected to be always available in pharmacies. In this study, only the lowest price of generic (LPG) medicines have been taken into account, not the brand or brand-generic ones. To clear more, the list of selected medicines was presented in Table 2.

*Sampling and Data Collection*

To carry out this research, 30 public pharmacies and 30 private pharmacies from five cities were selected. According to the data provided by Iran Central Bank, Tehran, as the most privileged, and Zahedan, as the least privileged cities were selected. Then, based on clustered sampling technique, to cover different geographic regions, three more cities, namely Mashhad, Yazd, and Tabriz, were randomly chosen. The research sample included pharmacies and pharmaceutical centers which provided out-patient services in public (general) levels and had a specific place to dispense medicines to the patients. Therefore, specialized services were excluded. The first medical service-providers were the biggest out-patient pharmacies belonging to public hospitals in each city. The second samples were the smallest pharmaceutical service-providers in one of health centers located in the least privileged part of the city. These centers were selected by vice-chancellor for food and drug in medical universities of each province. Four more pharmacies were selected randomly in different parts of the cities. Pharmacies belonging to universities, social security organization, charities, and the armed forces were assumed as public ones. Having selected public pharmacies, the researchers chose the nearest private centers to the selected public pharmacies as the samples of private sectors.

In data collection phase, some appointments were made with pharmacy managers so that data collectors could collect the necessary information. Furthermore, As the WHO guidelines recommend, both retrospective and prospective methods were employed in patients sampling. For example, to measure some indicators related to rational use of medicines, prospective sampling was randomly carried out from among thirty patients who referred to the pharmacy. In order to achieve comparable data from different cities, uniformity in data collection was necessary. Therefore, data collectors were provided with some information, such as key medicines list, treatment guidelines, checklists, official letter of introduction to the local health authorities, and identification card. According to the WHO guideline, the selected patients were suffering from common diseases such as non-bacterial diarrhea, mild/moderate (outpatient) pneumonia, non-pneumonia acute respiratory tract infection, diabetes, asthma, and hyperlipidemia. It is worth mentioning that patients who referred for prenatal and postnatal care, the elderly and the children′s healthcare, and specialized counseling were excluded due to the difference in their treatments. The data were collected from April to November 2018.

*Data Analysis*

During the data gathering process, the non-qualified data were modified and the biased data were deleted. SPSS 24 was employed to analyze the data. Depending on proving data normality based on the Kolmogorov-smirnov test, Independent sample *t*-test and one-way ANOVA test were performed to test significant differences among various sectors with *P*-value <0.05 or <0.1.

## Results

*Access*

The data related to the availability of key medicines in public and private pharmacies, the percentage of dispensed medicines, and geographical availability of pharmacies in studied cities are provided in Table 3. Based on the results drawn from one-way ANOVA and Independent sample *t*-test, there was no significant difference in the availability of key medicines in various cities, nor between public and private pharmacies. In comparison with private pharmacies, public pharmacies had dispensed a higher percentage of prescribed medicines. However, in terms of geographical availability of pharmacies, private pharmacies must have enjoyed a better condition.

The medicines’ prices, both in private and public pharmacies, were not different in the studied cities. The prices of key medicines list were calculated and then compared with the average world prices. The median price for one medicine in Iran compared to its world price (MPR) was equal to 1.33, which is acceptable.

As Table 4 demonstrates, for mentioned chronic diseases or common acute infections, the cost of medicines for one period of standard treatment regimens based on STGs is lower than the wage of LPGW. Even though the cost of treatment in private sector is higher than those of public sector, the purchase of the medicines for a period of treatment is affordable in both sectors.

*Quality*

As mentioned, in this study, quality was evaluated by considering lack of expired medicines in pharmacies and the proper conditions in which medicines are kept. As Table 5 demonstrates, there were no expired medicines in selected pharmacies (except for one in a public pharmacy in Tabriz). Test results drawn from Independent sample *t*-test revealed that the public sector enjoyed a more favorable condition and made a significant difference with private sector regarding these two indicators, namely ″appropriately storing medicines″ and ″appropriately keeping medicines in dispensing area″. In addition, among public pharmacies, the adequate conservation conditions and handling of medicines in the storeroom and dispensing area in Zahedan is much poorer than those in other cities, while among private pharmacies, these two indicators were much worse in Tehran than in other cities.

*Rational use of medicines*

Concerning rational use of medicines, prescribing antibiotics and injectable medicines, prescribing drugs using their Generic names and also based on available key medicines in public sector were studied. The average number of medicines per prescription were examined both retrospectively and prospectively. In prospective approach, 30 patients referred to the centers were randomly selected and their prescriptions were examined. In accordance with the WHO′s instructions, retrospective approach was carried out only in public sector; here, we selected randomly 30 general prescriptions registered on previous days and examine them. The average number of medicines per prescription, both retrospectively and prospectively, in public pharmacies was 3.2. Besides, based on prospective sampling this indicator was 3.4 in private pharmacies, which made a significant difference with public pharmacies. Studies in public sector revealed that, on average, 33% of patients received antibiotics and 32% were delivered injectable medicines. Also, 77% of medicines were prescribed using their generic names and 25% of prescriptions were from key medicines list. It is worth mentioning that 92% of medicines delivered to the patients contained adequate label, and 96% of patients precisely knew how to take their medicines. In addition, there was no significant difference between private and public centers concerning appropriately labeling the medicines and informing the patients about medicine consumption. Table 5 demonstrates the results regarding the indicators of rational use of medicines in detail.

## Discussion

**Figure 1.**
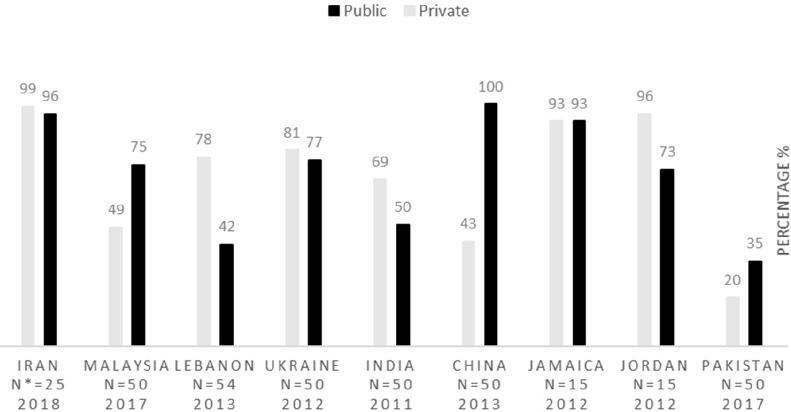
Availability of key medicines in Iran compared with other countries

This study intends to evaluate Iran′s pharmaceutical situation in terms of access, quality, and rational use of medicines indicators in accordance with the WHO′s level II instructions. Concerning accessibility, which is upon the government to guarantee that the essential medicines should be available in pharmacies and be affordable for vulnerable groups (2), the results of this study reveal that the lowest price of key generic medicines, both in public and private sectors, are optimally available in Iran. Since the WHO declares that the acceptable level of availability for key medicines is at least 80% (16), this indicator, in Iran, is higher than this acceptable level. Figure 1 demonstrates the key medicines availability in some countries. Three more studies carried out in 2003, 2007, and 2014 in Iran approve these findings; the availability of key medicines in these studies is reported to be higher than 90% (11) (12). These data are compared with that of other countries, including Malaysia (17), Lebanon (18), Ukraine (19), India (20), China (21), Jamaica (22), Jordan (23) and Pakistan (24).

*N: the number of key medicines

**Figure 2:**
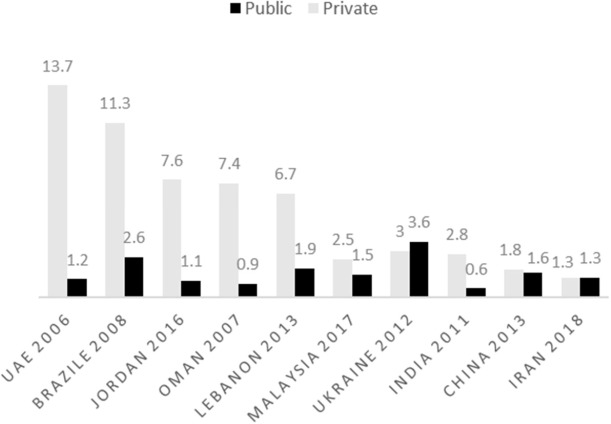
MPR in Iran compared with other countries

MPR indicator is a way with which the price of a medicine can be compared with that of other countries and its acceptable level is lower than 1.5 and 2 in public and private pharmacies, respectively (15). Figure 2 compares this indicator in Iran and other countries. The results of this chart indicate that medicine price, in public sector of Iran, enjoys an optimal condition, while this indicator in private sector in most countries is much higher than the acceptable level. Studies conducted in 2007 and 2014 confirm the optimal condition of MPR in Iran (12). These data are compared with that of other countries, including United Arab Emirates (25) , Brazil (26), Jordan (27), Oman (28), Lebanon (18), Malaysia (17), Ukraine (19), India (20), and China (21).

The results of the current research indicate that, in terms of affordability, Iran enjoys an optimal condition even if the medicines are not covered in insurance plans. To clarify this point, this indicator for Metformin without insurance coverage is 0.3 in Iran, 0.4 in Lebanon and Oman (18, 28), 0.5 in United Arab Emirates (UAE) (25), 0.6 in Jordan (27), 1.4 in Ukraine (19), and 2.1 in Jamaica (22). Similarly, this indicator for hypertension treatment with Captopril or Enalapril is 0.1 in Iran, 0.3 in China (29), 0.5 in Ukraine (19), 1.6 in Jordan and UAE (25, 27). Affordability for Asthma with inhaled Salbutamol is 0.4 in Iran and India (20); 0.3 in Oman (28), Lebanon (18), and Jordan (27); 0.5 in Ukraine (19); 0.6 in UAE (25) and China (29); and 1.6 in Brazil (26). Finally, this indicator for a seven-day treatment with Amoxicillin is reported 0.3 in Iran, 0.4 in Oman and UAE (25, 28), 0.5 in Ukraine (19), 0.6 in Jordan (27), 0.8 in India and China (20, 29), and 1.2 in Brazil (26). This achievement might root in Iran pharmaceutical policies such as price control, medicines distribution, and generic policies.

As it was mentioned earlier, rational use of medicines is one of the most challenging issues in medical policy making. Since policy makers have no complete control on the ″key prescribers″ and patients, controlling and optimizing the indicators in this field is very difficult (30). The WHO asserts that the acceptable average number of medicines per prescription is 2 or less. Prescribed antibiotics and injectable medicines, according to the WHO guidelines, should be maximally up to 30% and 10% for treated patients, respectively (31). The WHO instructions also maintains that the closer the percentage of prescription using the Generic name and key medicines lists to 100% is, the better it is (31). This study showed that the average number of medicines per prescription in public sector was 3.2, which is in parallel with previous studies conducted in 2003, 2011, and 2013 in Iran (11, 32, 33). Considering the findings of studies in other countries, they enjoy a better condition in this area. So, Iran needs more improvement regarding this indicator. These data are compared with that of other countries, including Jamaica (22), Jordan (23), Kuwait (34), India (35), United Arab Emirates (36), Saudi Arabia (37), Brazil (38), and China (39).

The percentage of patients for whom Antibiotics or injectable medicines are prescribed is another indicator demonstrating rational use of medicines because overconsumption of antibiotics brings about microbial resistance and injectable medicines increase risk of some complications. Figure 4 demonstrates the percentage of patients for whom antibiotics and injectable medicines have been prescribed is higher than other countries. These data are compared with that of other countries, including Jordan (23), India (35), China (39), Kuwait (34), Jamaica (22), Saudi Arabia (37), Brazil (40), and United Arab Emirates (36).

As it can be seen, injectable medicines consumption is much higher than the accepted level in the current study, as well as the studies conducted in 2003, 2011, and 2013 (11, 32, 33). There is a law in many public hospitals in UAE and Saudi Arabia stipulating that physicians are not allowed to prescribe an injectable medicine if its non-parenteral form is available (36, 37). Cultural factors and attitudes of both physicians and patients are important factors that may be affect this issue (41). Furthermore, poor-quality prescribing is taken by general practitioners more than specialists (42). However, in the case of number of items in a prescription and the percentage of antibiotics and injectable medicines in prescription developed countries, thanks to more optimal policy-making and governance, are in a more suitable condition (43).

**Table 1:** Summary list of indicators used in present study

	**dimensions**	**indicators**
1	Access	Availability of key medicines
		% of prescribed medicines dispensed
		Affordability of standard treatment regimens
		Median price ratio of key medicines
		Geographical accessibility to pharmacies
2	Quality	% expired medicines in pharmacies
Adequacy of conservation conditions and handling of medicines in dispensing facilities		
		Proper conditions and good handling of medicines in storerooms
3	Prescription and rational use of medicines	Average number of medicines per prescription (retrospective)
		Average number of medicines per prescription (prospective)
		% patients prescribed antibiotics in public health facilities
		% patients prescribed injectable medicines in public health facilities
		% medicines prescribed by generic name at public health facilities
		% prescribed medicines on the key medicines list at public health facilities
		% medicines adequately labelled at health facility dispensaries and private pharmacies
		% patients knowing how to take medicines at public and private pharmacies

**Table 2:** The key medicines list

	**Medicine**	**Dosage form**		**Medicine**	**Dosage form**
1	Fluoxetine	20 mg cap	14	Atorvastatin	20 mg tab
2	Amoxicillin	500 mg cap	15	Sodium valproate	200 mg tab
3	Metoprolol	50 mg tab	16	ASA	80 mg tab
4	Losartan	25 mg tab	17	Prednisolone	5 mg tab
5	Ceftriaxone	1 g vial	18	Metformin	500 mg tab
6	Ciprofloxacin	500 mg tab	19	Folic acid	1 mg tab
7	Co-trimoxazole	480 mg tab	20	Mebendazole	100 mg tab
8	Alprazolam	0.5 mg tab	21	Povidone iodine	10% solution 250 mL
9	Ibuprofen	400 mg tab	22	Oral rehydration salts (ORS)	1 sachet
10	Glibenclamide	5 mg tab	23	Normal Saline	500 ml IV Fluid
11	Omeprazole	20 mg cap	24	Captopril	25 mg tab
12	Acetaminophen	25 mg/mL syrup	25	Dexamethasone	8 mg Injection
13	Salbutamol	0.1 mg/dose inhaler			

**Table 3:** Access to key medicines

	Tehran		**Zahedan**		**Tabriz**		**Mashhad**		**Yazd**	
	**Public**	**Private**	**Public**	**Private**	**Public**	**Private**	**Public**	**Private**	**Public**	**Private**
% key medicines available	96	99	96	100	93	97	99	100	96	100
% of prescribed medicines dispensed	98	85	98	99	95	75	97	90	95	86
Geographical accessibility to pharmacies %	60	95	88	77	55	89	48	70	91	71
Median price ratio of key medicines*	1.33	.33	1.33	1.33	1.33	1.33	1.33	1.33	1.33	1.33

*MPR: ratio of median local unit price to international reference unit price

- Based on, the data presented in the table have significant differences among different cities with *P*-value <0.05.

**Table 4:** The affordability of standard treatment regimens

	Reimbursed*		**Not reimbursed**	
	**Public**	**Private**	**Public**	**Private**
**Diabetes**				
Glibenclamide 5 mg tab BD	0.03	0.07	0.07	0.1
Metformin 500 mg tab TDS	0.08	0.1	0.28	0.31
Gliclazide 80 mg tab QD	0.04	0.08	0.1	0.15
Insulin NPH OR Regular 2 Vial+15 syringe	0.33	0.39	1.3	1.35
**Asthma**				
Salbutamol 100mcg/dose Inhaler	0.13	0.19	0.35	0.42
Beclomethasone 250mcg	0.17	0.24	0.45	0.52
**Blood pressure**				
Atenolol 50 mg QD	0.02	0.04	0.08	0.1
Captopril 25 mg BD	0.04	0.06	0.1	0.13
Amlodipine 5 mg QD	0.04	0.06	0.08	0.1
Enalapril 10 mg BD	0.15	0.22	0.15	0.22
Losartan 50 mg QD	0.04	0.06	0.1	0.13
Hydrochlorothiazide 25 mg QD	0.02	0.04	0.06	0.08
**Hyperlipidemia**				
Simvastatin 20 mg QD	0.07	0.13	0.16	0.23
**Upper respiratory tract infectious**				
Amoxicillin 500 mg tab TDS for 7 days	0.08	0.14	0.2	0.3
Ciprofloxacin 500 mg tab BD for 7 days	0.05	0.1	0.1	0.2
**Moderate pneumonia (without hospitalization)**				
Azithromycin cap 250 mg 6 tab/cap	0.07	0.14	0.18	0.25
For children: Azithromycin suspension 200 mg/5mL	0.1	0.17	0.17	0.23

* under the coverage of social security organization and Iran health insurance organization.

**Table 5:** The Quality of medicines

	**Tehran**		**Zahedan**		**Tabriz**		**Mashhad**		**Yazd**	
	**Public**	**Private**	**Public**	**Private**	**Public**	**Private**	**Public**	**Private**	**Public**	**Private**
medicines expired in outlets (%)	0	0	0	0	1	0	0	0	0	0
Adequacy of conservation conditions and handling of medicines in dispensing facilities (%)	88	68	76	73	82	79	92	71	76	82
Proper conditions and good handling of medicines in storerooms (%)	94	71	77	74	84	86	92	71	89	80

Based on Independent sample *t-test*, the data of condition and handling of medicines have significant differences between public and private sectors with *P*-value <0.1.

**Table 6:** The status of rational use of medicines indicators

	**Tehran**		**Zahedan**		**Tabriz**		**Mashhad**		**Yazd**	
	**Public**	**Private**	**Public**	**Private**	**Public**	**Private**	**Public**	**Private**	**Public**	**Private**
Average number of medicines per prescription (prospective)	3	3.7	3.5	3.8	2.9	3.4	3.5	2.8	3.1	3.6
Average number of medicines per prescription (retrospective)	3		3.4		2.8		3.3		3.2	
% patients prescribed antibiotics in public facilities	32		43		17		26		45	
% patients prescribed injections in public facilities	37		34		19		40		32	
% medicines prescribed by generic name at public facilities	75		92		61		76		79	
% prescribed medicines on the key medicines list at public facilities	26		32		17		24		25	
% medicines adequately labelled at public and private facilities	97	91	100	99	100	99	92	81	79	79
% patients knowing how to take medicines at public and private facilities	99	98	99	99	100	100	99	97	87	93

**Figure 3:**
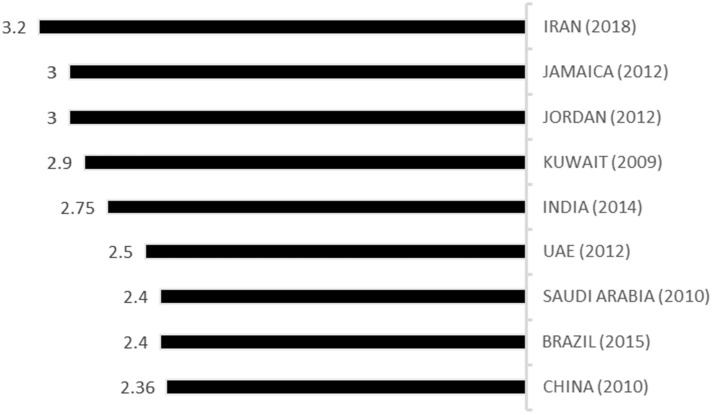
Average number of medicines per prescription in Iran compared with other countries

**Figure 4:**
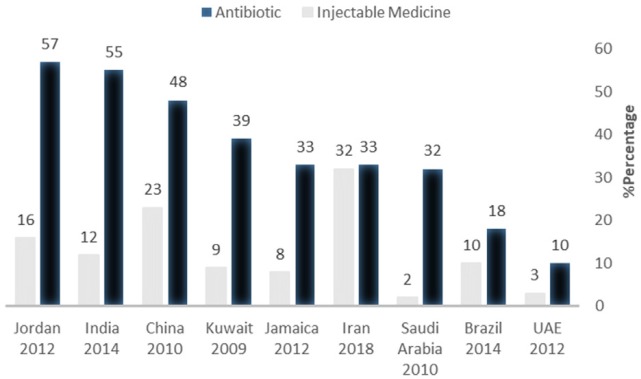
Percentage of patients prescribed antibiotics and injectable medicine in some countries

**Figure 5:**
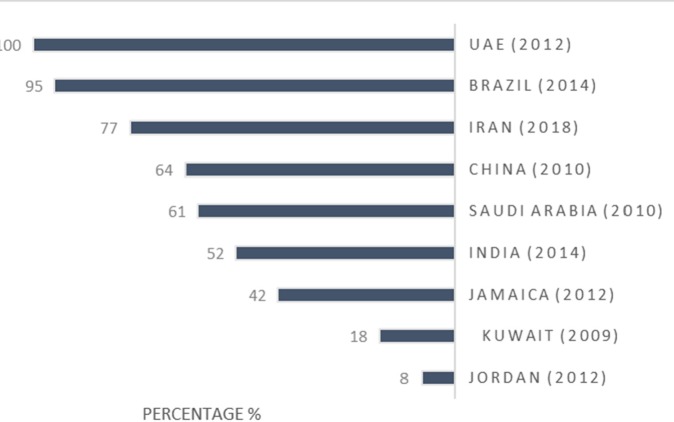
The percentage of medicines prescribed by Generic name (INN)

The percentage of medicines prescribed by generic name is another indicator showing rational use of medicines. Prescribing and using generic medicines which have as much efficacy as brand ones is more favorable for governments because it contributes to saving more money in public resources. However, pharmaceutical companies′ advertisements and families′ raise of income push physicians and people to prescribe and consume brand medicines. Figure 5 shows percentage of medicines prescribed by generic name in surveyed countries. These data are compared with that of other countries, including United Arab Emirates (36), Brazil (40), China (39), Saudi Arabia (37), India (35), Jamaica (22), Kuwait (34), and Jordan (23).

Although in UAE the public sectors are required to prescribe medicines with their generic names (36), according to survey conducted in private centers there, prescription indicator for generic medicines amounted to 7% (44).

*Conclusion and Recommendation*

This study intended to evaluate Iran pharmaceutical situation in terms of some indicators such as availability, affordability, quality of medicines and rational use of medicines indicators. This research revealed that Iran was in a good condition regarding availability and affordability of key medicines both in public and private sectors. Likewise, by inspecting whether there were any expired medicines and whether they were kept and stored in suitable conditions in pharmacies, the quality of medicines and pharmacy practices was approved. However, the results of this study showed that Iran is not in a favorable condition regarding rational use of medicines indicators, such as the average number of medicines, antibiotics, and injectable medicines per prescription. To improve the condition, it is recommended that some incentive and punitive measures for physicians to prescribe generic medicines, and pharmacists be encouraged to generic substitution. Also, physicians be obliged to follow the clinical guidelines. In addition, the electronic systems which monitor prescription behavior, and public education are recommended. It must be noted that insurance organizations, as the service purchasers, play a key role in directing physicians and promoting rational use of medicines indicators. Hence, to promote the pharmaceutical system′s performance, the followings are recommended.

In order to more efficient management of access, it is recommended that the list of essential medicines for different circumstances be classified and prescriptions directed to this list. 

Although affordability for different types of medicines in Iran is mainly better than that in other countries, more incentive policies can be considered for generic medicine may improve rational use of medicines and lead to more efficient use of resource.

Due to the necessity of continuous and accurate observation of drug utilization, developing “personal electronic health record” can be an effective measure to promote rational use of medicines indicators. 

*Limitations*

Every study has limitations. In this study, the relatively small sample size in each facility may decreases the reliability of the inter-facility comparison. In addition, the methodological pitfall regarding the wage of the lowest paid unskilled government worker is considered for unemployed persons, who have a daily wage less than the afore-mentioned amount, and who have more than one patient in a family. 
